# Complete genome sequence of *Salmonella enterica* serovar Sendai shows H antigen convergence with *S.* Miami and recent divergence from *S.* Paratyphi A

**DOI:** 10.1186/s12864-019-5798-7

**Published:** 2019-05-22

**Authors:** Ye Feng, Enze Lin, Shengmei Zou, Chyi-Liang Chen, Cheng-Hsun Chiu

**Affiliations:** 1 Sir Run Run Shaw Hospital, Zhejiang University School of Medicine, Hangzhou, China; 2 Institute for Translational Medicine, Zhejiang University School of Medicine, Hangzhou, China; 3 Key Laboratory of Microbial Technology and Bioinformatics of Zhejiang Province, Hangzhou, China; 4 Molecular Infectious Disease Research Center, Chang Gung Memorial Hospital, Chang Gung University College of Medicine, Taoyuan, Taiwan; 5 Division of Pediatric Infectious Diseases, Department of Pediatrics, Chang Gung Memorial Hospital, Chang Gung University College of Medicine, Taoyuan, Taiwan

**Keywords:** CRISPR, Host restriction, Pseudogene, Recombination, Serovar

## Abstract

**Background:**

*Salmonella enterica* consists of over 2500 serovars and displays dichotomy in disease manifestations and host range. Except for the enrichment of pseudogenes in genomes for human-restricted serovars, no hallmark has been identified to distinguish those with host-generalist serovars. The serovar Sendai is rare and human-restricted. Notably, it exhibits an O, H antigen formula as the host-generalist serovar Miami.

**Results:**

We sequenced the complete genomes of the two serovars Sendai and Miami. Analysis at both nucleotide identity and gene content level demonstrates the same high degree of similarity between Sendai and Paratyphi A, but their distinct CRISPR spacers suggests a recent divergence history. A frameshift mutation occurred in *rfbE* for the entire lineage of Paratyphi A but not in Sendai, which may explain their distinct O antigens. The nucleotide sequence of Miami’s *fliC* is nearly identical to Sendai’s. The incongruent phylogeny of this gene with that of the adjacent genes suggests a recombination event responsible for Sendai and Miami possessing the same H antigen. Sendai’s even greater number of pseudogenes than that of Paratyphi A and Typhi indicates its undergoing continued genomic degradation. The phylogenetically distinct human-restricted serovars/strains share pseudogenes with the same inactivation mutations, therefore suggesting that recombination may have occurred and have been facilitated by their overlap in niches.

**Conclusions:**

Analysis of Sendai’s genome and comparison with others reflect the finer evolutionary signatures of *Salmonella* in the process of niches changing from facultative to obligate parasite.

**Electronic supplementary material:**

The online version of this article (10.1186/s12864-019-5798-7) contains supplementary material, which is available to authorized users.

## Background

*Salmonella enterica* is one of the world’s primary causes of foodborne illness. This is an extremely diverse species: According to the Kauffmann-White-Le Minor serotyping scheme, 46 O serogroups and 114 H antigens have been identified in its genome, and their various combinations make up over 2500 serovars [1]. While most cause self-limiting gastrointestinal diseases in a wide range of mammalian hosts, a few serovars, such as Typhi and Paratyphi A, specifically infect humans and elicit typhoid, paratyphoid or enteric fever, which are severe infections of the reticuloendothelial system with high rates of complication and mortality [2, 3].

Although this dichotomy in disease manifestation and host range is well recognized, little progress has been achieved in exploring the genetic determinants responsible for these two distinct phenotypes until recently. The typhoid toxins CdtB and PltAB, which are encoded by typhoidal serovars but absent from most non-typhoidal serovars, have been shown to be unable to bind glycosylated surface glycoprotein receptors in non-human cells [4]. A Rab32-dependent pathogen-restriction mechanism has also been identified that limits the growth of the typhoidal serovars within macrophages of non-permissive animals; whereas broad-host *Salmonella* serovars are able to proteolytically target Rab32 with a type III secretion effector GtgE, which is absent from typhoidal serovars [5]. These findings suggest the multifactorial nature of host specificity, and that there are likely several mechanisms responsible for it. In the meantime, the hallmark of the host-restricted serovars, as compared to their host-generalist relatives (such as Typhimurium and Enteritidis), is their accumulation of a large number of pseudogenes [6–11]. This is likely due to their selection during their colonization of the intestine (which may stimulate host immune responses), or genetic drift since intestinal colonization is not required to sustain a systemic infection, or a combination of both [6].

The serovar Sendai is also a strict human serovar and elicits enteric fever. It is thought to be closely related to Paratyphi A based on restriction fragment length polymorphism (RFLP) pattern [2], but the exact genetic distance between the two serovars is unclear. Biochemically, the two serovars both produce little H_2_S and fail to ferment tartrate or grow in citrate medium, but Sendai is distinctive in being able to ferment xylose [2]. Serologically, Sendai resembles Paratyphi A in having the O antigens 1 and 12, but differs in expressing O antigen 9 instead of 2. They both have the Phase 1 flagellar antigen, a, and the Phase 2 antigens, 1 and 5, but the Phase 2 antigens are not usually expressed in Paratyphi A. Interestingly, Sendai has the same antigenic profile as Miami, another serovar which is reported to be closely related to Panama genetically, as revealed by RFLP [2]. Clinically, Miami has been isolated from patients with acute gastroenteritis or similar infections rather than enteric fever [12]. In terms of host range, based on the literature Miami has been isolated from both warm- and cold-blooded animals [13].

Currently, complete genomes of Sendai and Miami are not yet available in public databases, but comparisons between them and other serovars may benefit the understanding of *Salmonella*’s evolution in at least the following two points. The first is host restriction. Sendai itself has little clinical research value due to few infection cases being caused by this serovar. However, as the only serovar, aside from Typhi and Paratyphi A, that is solely restricted to humans, genomic analysis of this group may afford rare insight into the process of convergent human adaptation. The second is the lack of corresponding antigenic similarity and phylogeny among Sendai, Miami and Paratyphi A. It has long been known that a few serovars such as Paratyphi B and Newport exhibit polyphyletic behavior [14, 15]. The most obvious explanation for the polyphyletic origin of these serovars involves a mechanism by which the surface antigens that define the serovar are transferred to a different genomic context. This explanation may also apply to the situation concerning Sendai and Miami, but the molecular details of the horizontal transfer among the three serovars remains unknown. In this study, we sequenced the complete genomes of Sendai and Miami and attempted to address the above two issues.

## Results and discussion

### The genetic relationship between Sendai, Miami and Paratyphi A

The complete genomes of Sendai strain BAA1672 and Miami strain BAA1586 were sequenced. Sendai carried a plasmid which was 81,125 bp long and possessed two replicons, IncFII and IncFIB. Miami carried a 94,044 bp IncFII(S) plasmid. Neither of the two plasmids carried the *spv* operon or acquired antimicrobial resistant genes.

**Fig. 1:**
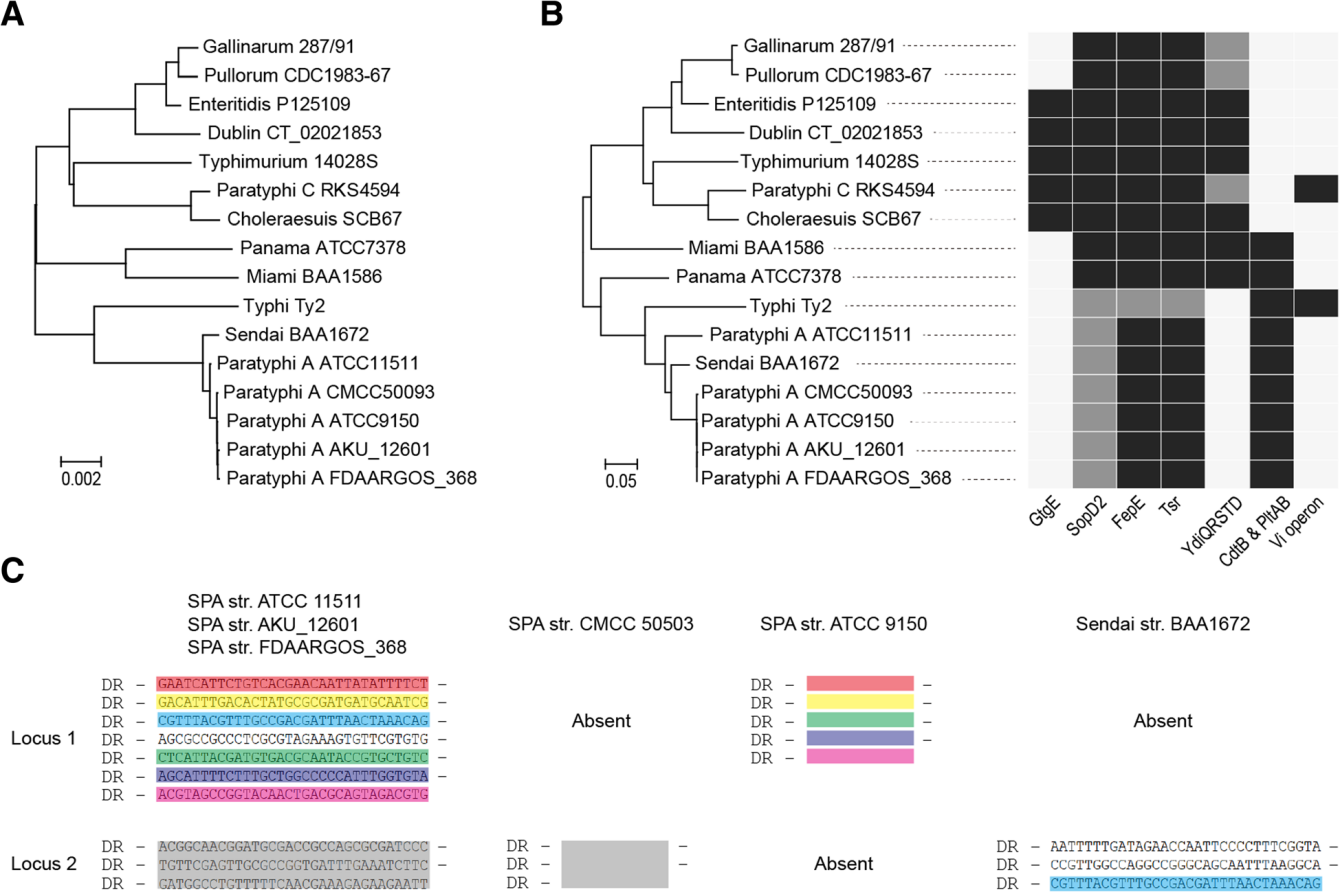
The genetic relationships between Sendai and Paratyphi A. **a** The neighbour-joining tree based on the genetic distance which is defined as 100% minus ANI. **b** The neighbour-joining tree based on the gene content matrix. The right heatmap shows the presence/absence of known host-related virulence factors in these serovars. Black, presence; white, absence; grey, pseudogene. **c** CRISPR spacers in Paratyphi A and Sendai. The *Salmonella* genome contains two CRISPR loci. DR represents the conserved direct repeats of the two CRISPR loci, which are 29 bp long and possess the consensus sequence, 5′-CGGTTTATCCCCGCTGGCGCGGGGAACAC-3′. Spacers of the same colour indicate identical sequences. Paratyphi A strain ATCC 11511, AKU_12601 and FDAARGOS_368 share the same CRISPR loci. Paratyphi A strain CMCC 50503 lacks Locus 1 and has the identical Locus 2 as the above three Paratyphi A strains. Paratyphi A strain ATCC 9150 lacks Locus 2 and its Locus 1 lacks two internal spacers as compared with the other Paratyphi A strains. Sendai lacks Locus 1; its Locus 2 shares one spacer with Paratyphi A (in light blue) but the other spacers were different. SPA: *S.* Paratyphi A

The chromosomes of the two strains had lengths of 4,483,399 bp and 4,639,823 bp, respectively. We compared them with the representative genomes of other *Salmonella* serovars by average nucleotide identity (ANI) (Table 1). The phylogenetic tree based on pairwise ANI revealed a close relationship between Sendai and Paratyphi A (Fig. 1a): The ANI between Sendai and the last common ancestor of the five Paratyphi A strains was 99.887%. We compared the similarity with that of two other pairs of serovars. One pair was Choleraesuis and Paratyphi C, with a pairwise ANI 99.766%. They both revealed the same O, H antigen formula of 6,7:c:1,5, but were thought to have diverged due to a host shift: Choleraesuis has adapted to infect swine, whereas Paratyphi C prefers to infect humans. The other pair was Galinarum and Pullorum, with a pairwise ANI 99.813%. They both have the O, H antigen 1,9,12:-:-, and both use poultry as their unique host while exhibiting slightly different infection symptoms; they are considered to be different biovars within the same serovar. Sendai and Paratyphi A’s having a much shorter genetic distance between them suggests their very recent divergent history, which is even shorter than that for *Salmonella* biovars. Meanwhile, Miami was indeed close to Panama (Fig. 1a), but they were considered to have diverged much earlier according to their relatively large genetic distance.

**Table 1:** The strains used for genome comparisons in this study

“O”-group	Serovar	O antigens	Phase 1 H antigens	Phase 2 H antigens	Host	Disease in humans	Strain	Pseudogenes	Accession no.	Reference
**A**	Paratyphi A	1,2,12	a	NA	Humans	Typhoid fever	ATCC 9150	242	NC_006511	[9]
							AKU_12601	242	NC_011147	[6]
							ATCC 11511	242	CP019185	[14]
							CMCC50093	246	CP011967	[40]
							FDAARGOS_368	285	CP023508	Not available
**B**	Typhimurium	1,4,5,12	i	1,2	Broad	Gastroenteritis	14028S	130	NC_016856	[41]
**C**_**1**_	Paratyphi C	6,7,	c	1,5	Humans^a^	Typhoid fever	RKS4594	285	NC_012125	[11]
	Choleraesuis	6,7	c	1,5	Pigs^a^	Gastroenteritis	SCB67	347	NC_006905	[42]
**D**	Typhi	9,12,Vi	d	NA	Humans	Typhoid fever	Ty2	254	NC_004631	[8]
	Dublin	1,9,12	g,p	NA	Cattle^a^	Gastroenteritis	CT_02021853	201	NC_011205	[43]
	Enteritis	1,9,12	g,m	NA	Broad	Gastroenteritis	P125109	140	NC_011294	[7]
	Gallinarum	1,9,12	NA	NA	Fowl	Asymptomatic	287/91	337	NC_011274	[7]
	Pullorum	(1),9,12	NA	NA	Fowl	Asymptomatic	CDC1983–67	327	NC_022221	[10]
	Panama	1,9,12	l,v	1,5	Broad	Gastroenteritis	ATCC 7378	176	CP012346	[44]
	Miami	1,9,12	a	1,5	Broad	Gastroenteritis	BAA1586	186	CP023468	This study
	Sendai	1,9,12	a	1,5	Humans	Typhoid fever	BAA1672	331	CP023470	This study

^a^Adapted, but no completely restricted

### Identification of gene content specific for typhoidal serovars

A comparison of gene content also revealed a close relationship between Sendai and Paratyphi A. We ascertained and compared all of Sendai’s chromosomal genes with the other genomes, determining that up to 99% of Sendai’s genes were present in Paratyphi A, with this rate being below 98% for the other serovars. We also constructed the pan-genome for *S. enterica* and measured the presence and absence of each gene in the analyzed genomes. A neighbor-joining tree thus constructed indicated that Sendai was even more closely related to Paratyphi A strain ATCC 11510 than the other Paratyphi A strains (Fig. 1b), suggesting that Sendai and Paratyphi A had shared the same genetic repertoire not long before. Meanwhile, Miami was still more closely related to Panama than to Sendai in terms of its genetic content.

From the pan-genome analysis, only 28 genes were found to be present in Sendai but absent from Paratyphi A (Additional File 1). The 28 genes were also present in other *Salmonella* serovars, and therefore Sendai possessed few of its serovar-specific genes. No genes were found to be shared only by Sendai, Typhi and Paratyphi A and C, suggesting that the typhoidal pathovars were not defined by the presence of shared virulence genes that were absent from non-typhoidal serovars.

The host adaptation of Typhi has been associated with horizontal gene transfer events (*cdtB*, *pltAB* and Vi operon), deletion of genes (*ydiQRSTD* and *gtgE*) and pseudogene formation (*sopD2*, *tsr* and *fepE*): 1) *cdtB* and *pltAB* encode typhoid toxins [4]; 2) inactivation of *gtgE* and *sopD2* boosts the Rab32-dependent host defense pathway that is critical for killing Typhi in macrophages from non-permissive animals [5, 16]; 3) inactivation of *ydiQRSTD* and *tsr* can repress T3SS-1 gene expression compared to zoonotic non-typhoidal serovars and therefore moderate intestinal inflammation [17]; 4) the polysaccharide encoded by Vi operon in Typhi and the long O-antigen chains led by inactivation of *fepE* in Paratyphi A both inhibit complement activation and moderate intestinal inflammation. These genes in Sendai were the same as in Paratypi A but different from Typhi (Fig. 1b). Notably, several serovars showed a somewhat similar pattern of these host-associated determinants to Typhi and Paratyphi A, including Gallinarum/Pullorum, a poultry-restricted serovar, Paratyphi C, a human-adapted serovar, and Panama and Miami, which are usually considered as broad-host serovars. These findings again suggest that the virulence properties shared by typhoidal serovars may be acquired through convergent evolution instead of vertical inheritance.

### Comparison of CRISPR loci between Sendai and Paratyphi A

Next, we analyzed the clustered, regularly interspaced short palindromic repeat (CRISPR) loci of the Sendai-Paratyphi A clade. The CRISPR system is characterized by 24–47 bp DNA direct repeats (DRs), separated by variable 21–72 bp sequences called “spacers” [18]. These spacers are acquired from foreign mobile genetic elements (MGEs, e.g. plasmids and bacterial phages) and integrated in a polarized fashion into the CRISPR array in bacteria and archaea. Subsequently, the CRISPR array(s) can be transcribed and processed into small CRISPR RNA molecules, which then, guide Cas (CRISPR-associated) proteins for sequence-specific recognition and degradation of the invader MGEs. Thus CRISPR-Cas systems provide acquired, heritable and adaptive immunity to bacteria and archaea against both viral and plasmid invasion [19, 20]. Micro-variations of the spacer content were found to be below the serotype level, and this polymorphism was strongly correlated with the phylogeny revealed by both serotyping and MLST [21], making it possible to carry out subtyping within prevalent serovars using CRISPRs.

Two CRISPR loci were separated by less than 20 kb in the *Salmonella* genomes. Three of the five analyzed Paratyphi A strains had identical spacer sequences for the two CRIPR loci, whereas the other two Paratyphi A strains lacked several spacer units within one locus and/or lack an entire locus (Fig. 1c). Sendai lacked Locus 1; its Locus 2 shared one spacer with Paratyphi A, but the other two spacers were absent from the analyzed Paratyphi A strains. We compared the two spacers in the NCBI nucleotide collection (nr/nt) database using a sequence similarity search (identity cutoff 90%), and obtained no hits. Miami possessed two CRISPR loci, both of which were different from those of Sendai and Paratyphi A.

It has been found that CRISPR spacers can target not only the sequences of exogenous MGEs but also those of chromosomal housekeeping genes of related species [22]. This suggests CRISPR can effectively build barriers to gene flow, thereby resulting in microbial speciation. The genetic barrier formed by CRISPRs is thought to play a role more over short time scales (intra-species) than over longer evolutionary time scales (inter-species) [23, 24], which is in line with differences in CRISPR spacers often found among subspecies. Due to the polarized spacer acquisition that has its ending spacer representing the recently encountered MGEs, the CRISPR locus can be used to reconstruct the history of past infection of MGEs. Furthermore, the generation of spacers shows a strong bias toward the frequently encountered MGEs rather than the full spectrum of the exogenous DNA [24]. Consequently, the different CRISPR spacers between Sendai and Paratyphi A may indicate the recently diverging niches of the two serovars, which would lead to their ultimate genetic divergence.

### The genetic basis of the O, H antigens in Sendai, Miami and Paratyphi A

**Fig. 2:**
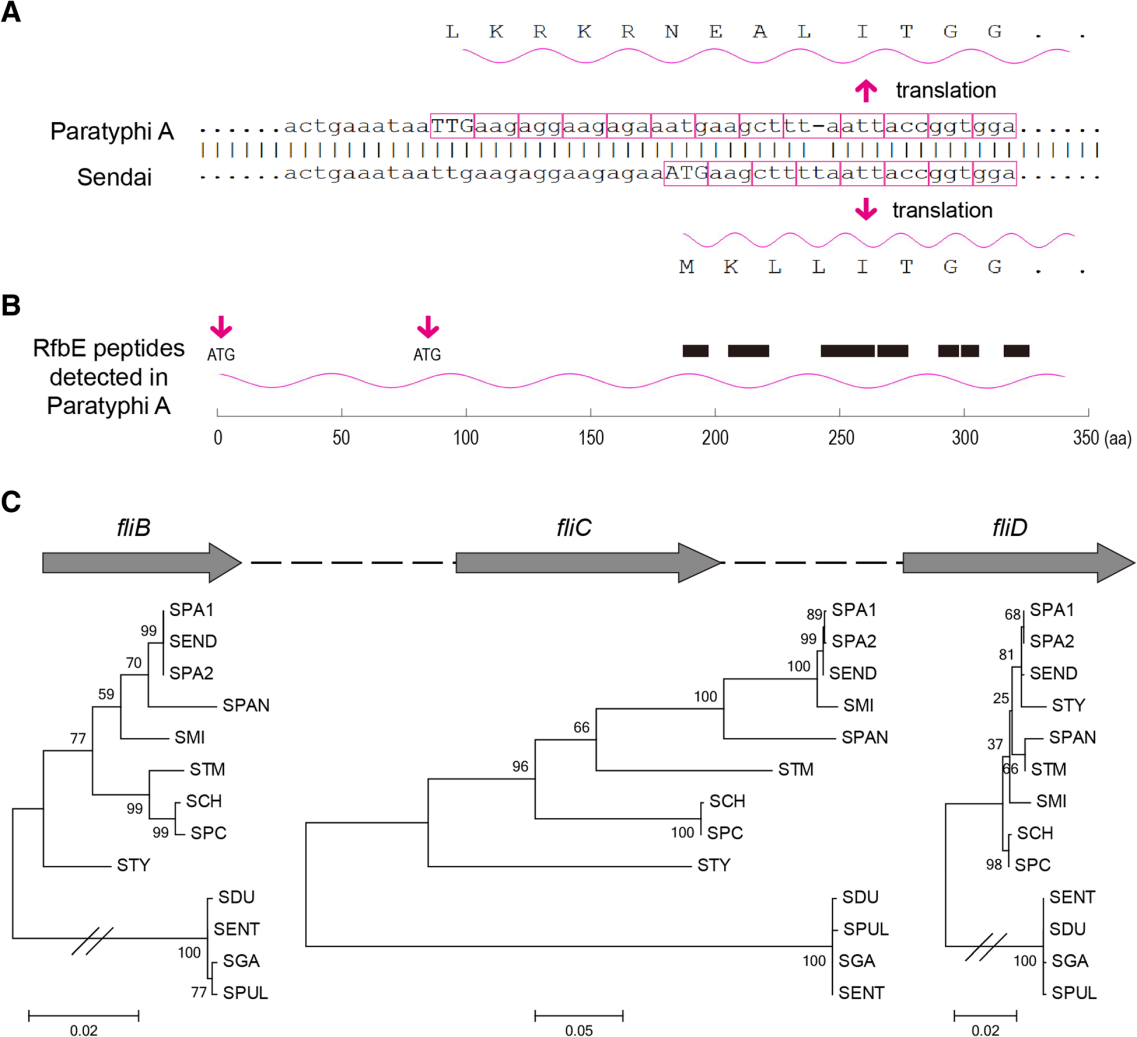
The molecular basis of O, H antigen formula for Sendai, Miami and Paratyphi A. **a** A frameshift mutation occurred in *rfbE* in the entire lineage of Paratyphi A, but not in Sendai. Consequently, Paratyphi A needs to recruit a different start codon to initiate translation. **b** Seven different peptides were detected for RfbE in Paratyphi A by proteomic analysis (see details in Additional File 2), with their positions marked by black boxes. The position of possible start codons (ATG) were marked by red arrows. **c** Incongruent phylogeny between *fliB*, *fliC* and *fliD*. For *fliC*, Miami is nearly identical to Paratyphi A and Sendai; but for *fliB* and *fliD*, Miami is relatively remote. SPA1, *S.* Paratyphi A ATCC 9150; SPA2, *S.* Paratyphi A ATCC 11511; SEND, *S.* Sendai BAA1672; SMI, *S.* Miami BAA1586; SPAN, *S.* Panama ATCC 7378; STM, *S.* Typhimurium 14028S; SCH, *S.* Choleraesuis SCB67; SPC, *S.* Paratyphi C RKS4594; STY, *S.* Typhi Ty2; SDU, *S.* Dublin CT_02021853; SPUL, *S.* Pullorum CDC1983–67; SGA, *S.* Gallinarum 287/91; SENT, *S.* Enteritis P125109

Given Sendai’s and Paratyphi A’s highly similar genomes, we next explored why the two serovars possessed different O antigens. It is known that the *Salmonella* serovars of Serogroups A and D share the same minor O antigens and that both proceed through the identical intermediate period during O-antigen synthesis, at which point the conversion of a paratose sugar to a tyvelose residue by the product of the *rfbE* locus generates the group D's O antigen [25]. The sequence of the entire *rfbE* locus was nearly identical in Paratyphi A and Sendai, with a 1-bp deletion occurring in the former but not in the latter (Fig. 2a). Next we performed a quantitative proteomic experiment and found that RfbE in Paratyphi A expressed in both LB and SPI-2-mimicked medium [26], with a comparable expression level to that in Typhimurium (Fig. 2b; Additional File 2). This suggests that Paratyphi A may recruit a further upstream start codon or the downstream 72th codon (ATG) to initiate the translation, resulting in a 5-aa longer or 71-aa shorter RfbE than the other *Salmonella* serovars.

The other question is why Miami shares the same Phase 1 H antigen a as Sendai and Paratyphi A rather than Panama’s l,v antigen or Typhi’s d antigen. The Phase 1 H antigen, including the internal surface-exposed and antigenically variable portion of the flagellar filament, is encoded by *fliC* [27, 28]. Within the flagellar biosynthesis operon, Miami was not clustered with Sendai and Paratyphi A for *fliB* and *fliD*, which was somewhat consistent with the whole-genome-based phylogeny (Fig. 2c). However, *fliC*’s sequence was nearly identical among the three serovars, which might have led to their having the common H antigen. The differences in phylogenetic topology between *flic*, *fliB* and *fliD* suggests the acquisition of *fliC* through recombination in Miami.

### Comparison of pseudogenes in human-restricted serovars

Here, we re-annotated and compared the pseudogenes from among the three human-restricted serovars. To focus on the pseudogenes whose inactivation may be related to the host restriction, only those genes which retained intact open reading frames in Enteritidis strain P125109 (broad host) were analyzed (Additional File 3). Sendai and Typhi strain Ty2 had 185 and 132 pseudogenes, respectively, whereas the two Paratyphi A strains, ATCC 9150 and ATCC 11511, respectively possessed 135 and 139 pseudogenes. In contrast, Miami possessed 51 pseudogenes.

**Fig. 3:**
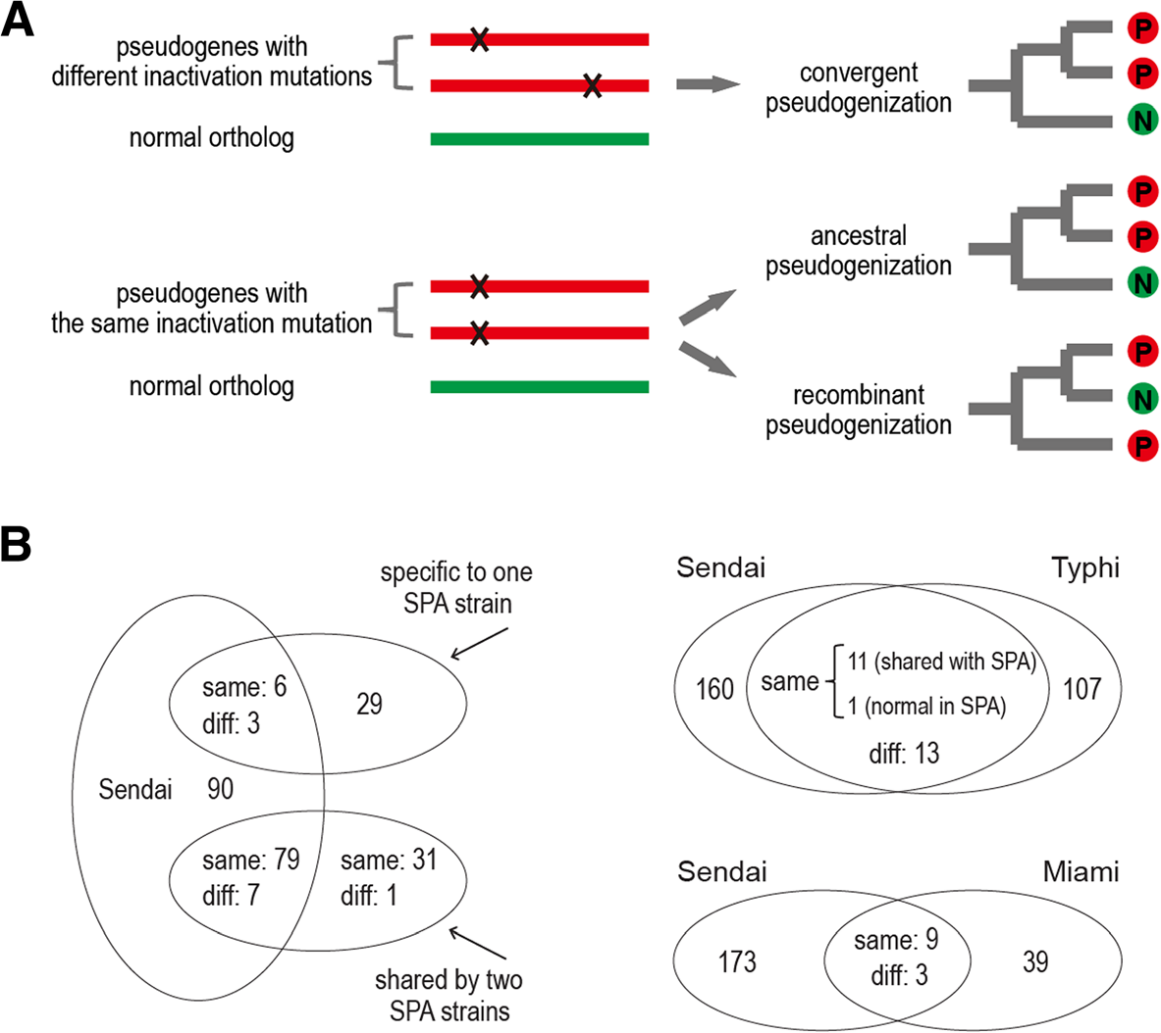
The compositions of the pseudogenes in Sendai, Paratyphi A, Typhi and Miami. **a** Schematic illustration of the different causes of pseudogenes shared between *Salmonella* serovars. The shared pseudogenes with different inactivation mutations are attributed to convergent evolution. Pseudogenes carrying the same inactivation mutations can further be attributed to two causes: 1) the ‘ancestral pseudogenization’ explains the strains that are phylogenetically clustered together and carry the same pseudogenes; and 2) the ‘recombinant pseudogenization’ explains the strains that are phylogenetically separated but carry the same pseudogenes. **b** The number of specific and shared pseudogenes between Sendai, Paratyphi A, Typhi, and Miami. The shared pseudogenes were further divided into other two groups, with ‘Same’ and ‘Diff’ representing the same and different inactivation mutations. SPA: *S.* Paratyphi A

We divided the pseudogenes into distinct categories in terms of their causes and relative ages. Strain-specific pseudogenes were deemed to have occurred more recently. Shared pseudogenes were further attributed to three causes according to their inactivating mutations and the phylogeny of the compared strains (Fig. 3a). Disparate inactivating mutations probably resulted in convergent loss, which occurred following divergence between the serovars/strains. The situation for the same inactivating mutations is more complex: They can occur in ancestral pseudogenes inherited from a common ancestor and pseudogenized prior to serovar/strain divergence, but they can also result from recombination, possibly arising following the initial divergence. Distinguishing between ancestral and recombinant pseudogenes depends on the circumstances.

Sendai and Paratyphi A shared 95 pseudogenes, 85 of which had the same inactivation mutations (Fig. 3b). Most of these were assumed to be ancestral pseudogenes, given the short divergence history between the two serovars. Interestingly, three genes had been inactivated in Sendai and only one Paratyphi A strain, which still remained intact in the other Paratyphi A strain. It is possible that the mutation initially occurred in Sendai, and a recombination event later carried the mutation from Sendai to one Paratyphi A strain. But it is also possible that the mutation had occurred in an ancestor of Paratyphi A, while Sendai delivered the intact fragment into one Paratyphi A strain via recombination.

In contrast, Sendai shared much fewer pseudogenes with Typhi than with Paratyphi A, a phenomenon consistent with their genetic distances. Of the 12 genes which Sendai shared with Typhi having the same inactivating mutations, one was intact in Paratyphi A, suggesting that its causal recombinant pseudogenization event had occurred following the divergence between Paratyphi A and Sendai (Fig. 3a). Meanwhile, Sendai and Miami also shared nine genes with the same inactivating mutations. Thus, recombination is not limited to the human-restricted serovars; typhoidal and non-typhoidal serovars can also infect humans simultaneously and exchange DNA.

Sendai, Typhi and Paratyphi A shared 26 pseudogenes (Additional File 4), which may result in or from convergent adaptation to a human host. Nevertheless, the inactivation of different genes within the same pathway will often result in a similar loss of function; thus, the true contribution of pseudogene formation to phenotypic convergence among the three typhoidal serovars is likely underestimated when merely considering the shared pseudogenes. We observed that the number of serovar-specific pseudogenes of Sendai is greater than that of Typhi and Paratyphi A. Host selection should not be the sole explanation for pseudogene formation in Sendai, since Typhi and Paratyphi A are also host-restricted. Paratyphi A has been reported as being deficient in its ability to import new genes from outside species due to a closed pan-genome [29]. In combination with the accumulation of pseudogenes, the Paratyphi A genome is undergoing degradation. This ratchet effect is more evident in Sendai. As a rare serovar, Sendai may have a much smaller population, suffer a narrower population bottleneck, and behave more like an asexual organism. The according genetic drift makes it difficult to reverse the trend of gene loss; the rate of pseudogene formation may be greater.

## Conclusions

In present study we reported the complete genome of S. Sendai and compared it with that of other *Salmonella* serovars. While mutations in *rfbE* have given rise to a novel O antigen and, accordingly, a novel serovar, Paratyphi A, recombination in *fliC* renders Miami the same H antigen as Sendai. The O antigen is subject to intense selection by the host’s immune system, bacteriophages, and other environmental factors [30]. Whether the above mutation and recombination events affect the virulence and even the host range is unknown.

Furthermore, we discovered recombination to be an essential mechanism in the pseudogenes of human-restricted serovars, as proven by their having the same inactivation mutations in their shared pseudogenes. In fact, recombination events are probably not limited to pseudogenes but also occur within other genomic regions, since 23% of the genomes had undergone recombination between Typhi and Paratyphi A [31]. This extensive recombination rate was possibly responsible for their convergence on a human-restricted lifestyle, but it is also plausible that the two serovars followed independent paths towards host-restriction and the opportunity for recombination arose after they became isolated together within this shared niche. Regardless of the reason, however, the recombination frequency in typhoidal organisms should not be exaggerated. The rapid accumulation of pseudogenes is still ongoing, suggesting that Sendai, Paratyphi A and Typhi are on a similar trajectory of host adaptation and are suffering the associated population bottlenecks.

## Methods

### Bacterial strains and genomic sequencing

The Sendai strain BAA-1672 and Miami strain BAA-1586 were obtained from an American type culture collection (https://www.atcc.org/). The bacterial genomic DNA was extracted using a QIAamp DNA Mini Kit (Qiagen, CA, USA). For both strains, a pair-end library and a mate-pair library were constructed, with the average fragment size being 300 bp and 3000 bp, respectively. The pair-end libraries launched on an Illumina Miseq sequencer (Illumina, CA, USA), and the mate-pair libraries launched on an Illumina Hiseq2000 sequencer (Illumina, CA, USA). The CLC Genomics workbench v9.5 was used for a de novo assembly of Illumina reads (Qiagen, CA, USA). The relationships between contigs was determined by mapping the contig sequences against the genome of *S.* Paratyphi A ATCC9150 (accession number NC_006511.1) with MUMmer v3.22 [32]. Gaps between contigs were closed by PCR amplification and Sanger sequencing.

### Genome annotation and bioinformatics analyses

The genomic sequences of Sendai strain BAA-1672 and Miami strain BAA-1586 have been deposited in the NCBI GenBank database, with their accession numbers being CP023470/CP023471 (chromosome/plasmid) and CP023468/CP023469 (chromosome/plasmid), respectively. The accession numbers and basic biological attributes of other representative *Salmonella* strains used for genome comparison are listed in Table 1. The NCBI Prokaryotic Genome Annotation Pipeline (PGAP v4.2) was used for basic genomic annotation [33]. The determination of sequence type for legacy multi-locus sequence typing (MLST) and prediction of the antimicrobial resistance genes were conducted using the online service, BacWGSTdb [34]. The detection of CRISPRs was performed using the online service, CRISPR CasFinder (http://crispr.i2bc.paris-saclay.fr/Server/) [35].

The ANI was measured by FastANI v1.1 [36]. The pairwise genetic distance was defined as 100% minus ANI, and accordingly a distance matrix was obtained for the compared genomes. The relatedness between the strains was also evaluated based on gene content. Briefly, a pan-genome was constructed for the analyzed genomes using Roary v3.11.1 [37]: the minimum percentage identity for blastp (−i) was set as 90 (default, 95); and the other parameters were set as default. Then a binary matrix with the presence and absence of each gene in the pan-genome was obtained. The pairwise distance between the strains was defined as the number of diverse genes divided by the total number of genes and was calculated based on the above matrix. The neighbour-joining trees were built via MEGA7 software using the above pairwise distance matrices as the input [38].

For an analysis of each gene within the serovar determinant *ofb* and *fli* loci, multiple alignments of the orthologous genes were performed with the Muscle program in the MEGA7 software, and neighbour-joining trees were also constructed for these genes by MEGA7.

For an analysis of the pseudogenes, the orthologous genes of each pseudogene obtained from the NCBI PGAP annotation were further identified using the NCBI blastn program (blastall v2.2.26; nucleotide identity cutoff, 0.85). A manual determination on whether the genes were intact was performed: the genes within the Enteritidis strain P125109 were used as a reference; orthologous genes that contained frameshifts, nonsense mutations, truncations, or indels that altered 20% of the amino acid sequence in comparison with the reference sequences were treated as pseudogenes. The exact inactivation site was identified using GeneWise software v2.4.1 [39].

### iTRAQ-LC-MS/MS proteome analysis

Bacterial proteins were extracted with 0.1% SDS solution by sonication. Forty micrograms of protein extracts were reduced and alkylated with dithiothreitol and iodoacetamide, respectively, and digested with 1 μg of modified trypsin (Promega, Madison, WI) at 37 °C overnight. Then the peptides were labeled using TMT 10 plex Mass Tag Labeling Kits (Thermo Fisher Scientific, Waltham, MA, USA) according to the manufacturer’s instructions. The 2D-SCX-RP-LC experiment was performed on a Dionex Ultimate 3000 nanoflow HPLC (Dionex, Germering, Germany). The effluent of the online 2D LC was analyzed by a LTQ-Orbitrap hybrid mass spectrometer (Thermo Electron, Bremen, Germany). Raw MS files from the LTQ-Orbitrap were analyzed by Mascot v2.2.2 (Matrix Science Inc., Boston, MA) and MaxQuant v1.0.13.13.

## Additional files

Additional file 1: Genes present in Sendai but absent from Paratyphi A (XLSX 10 kb)

Additional file 2: Peptides of RfbE identified by proteomic sequencing (XLSX 13 kb)

Additional file 3: Orthologous relationship between *Salmonella* strains (XLSX 752 kb)

Additional file 4: 26 common pseudogenes shared by Sendai, Typhi and Paratyphi A (XLSX 10 kb)

## Abbreviations

ANI: Average nucleotide identity
Cas: CRISPR-associated
CRISPR: clustered regularly interspaced short palindromic repeat
DR: Direct repeat
MGE: Mobile genetic element
MLST: Multi-locus sequence typing
PGAP: Prokaryotic genome annotation pipeline
RFLP: Restriction fragment length polymorphism
SCH: *S*. Choleraesuis
SDU: *S*. Dublin
SEND: *S*. Sendai
SENT: *S*. Enteritis
SGA: *S*. Gallinarum
SMI: *S*. Miami
SPA: *Salmonella* Paratyphi A
SPAN: *S*. Panama
SPC: *S*. Paratyphi C
SPUL: *S*. Pullorum
STM: *S*. Typhimurium
STY: *S*. Typhi Ty2
